# Leptin Promoted IL-17 Production from ILC2s in Allergic Rhinitis

**DOI:** 10.1155/2020/9248479

**Published:** 2020-09-09

**Authors:** Yanhui Wen, Qingxiang Zeng, Xi Luo, Renqiang Ma, Yiquan Tang, Wenlong Liu

**Affiliations:** ^1^Department of Otolaryngology, Dongguan Songshan Lake Center Hospital, Dongguan, China; ^2^Department of Otolaryngology, Guangzhou Women and Children's Medical Center, Guangzhou Medical University, Guangzhou, China; ^3^Department of Otorhinolaryngology, Otorhinolaryngology Hospital, The First Affiliated Hospital, Sun Yat-sen University, Guangzhou, China

## Abstract

**Background:**

Interleukin-17 plays important roles in allergic diseases. Several studies proved that leptin promoted Th17 immune responses by inducing ROR*γ*t transcription. ILC2 is an important member of the early stage of immune response. Therefore, we aimed to explore the effect of leptin on the IL-17 production by ILC2 in AR in this study.

**Methods:**

Fifteen AR patients and fifteen healthy controls were enrolled. Serum leptin levels were measured, and their correlation with the frequency of IL-17+ ILC2 cells was analyzed using enzyme-linked immunosorbent assay (ELISA) and flow cytometry. ILC2 was stimulated by leptin, and the expression of IL-17, IL-5, and IL-13 was detected by ELISA. The correlated pathways were confirmed by real-time PCR.

**Results:**

We found that serum leptin and the frequency of IL-17-producing ILC2s in AR were significantly higher compared with those in controls. After being incubated with leptin, the frequency of IL-17+ ILC2 cells and IL-17 production from ILC2 was upregulated compared with that in controls. We also found that leptin induced ROR*γ*t and Ahr expression by ILC2. Moreover, leptin-induced IL-17-producing ILC2 concomitantly expressed IL-5 and IL-13.

**Conclusions:**

Our data provide preliminary evidence that leptin-induced IL-17 production from ILC2 cells is dependent on ROR*γ*t and Ahr expression and the blockade of leptin may be a promising target for the treatment of AR.

## 1. Introduction

Epidemiological studies showed that more than 500 million people were affected by allergic rhinitis (AR) worldwide, especially children [1]. In recent years, the incidence of AR in China has been also increasing significantly [2].

Interleukin-17, mainly secreted from Th17 cells, plays important roles in allergic diseases. For example, IL-17 was found in the lungs, sputum, and bronchoalveolar lavage fluids (BALF) of asthmatic patients [3, 4]. Moreover, the level of IL-17 is correlated with the severity of AR [4]. Previous studies also suggested that exogenous anti-IL-17 and anti-IL-23 antibodies can alleviate the symptoms, Th2 response, and serum IgE level in AR mice induced by ovalbumin [5–7].

ILC2 is widely distributed in adipose-associated lymphoid tissue, intestine, lung, and skin and is an important member of the early stage of immune response. Recent studies showed that ILC2s can produce IL-17 and express higher levels of retinoic-acid-receptor-related orphan receptor-*γ*t (ROR*γ*t), which regulates IL-17 transcription in various immune cells [8, 9].

Leptin is a 16 kDa nonglycosylated polypeptide encoded by the obese (ob) gene, which regulates energy homeostasis, neuroendocrine function, angiogenesis, hematopoiesis, and T cell activation and function [10]. Several studies proved that leptin promoted Th17 immune responses by inducing ROR*γ*t transcription in systemic lupus erythematosus and arthritis [11, 12]. Our previous data provide evidence that the upregulation of leptin promotes ILC2 responses in AR and this process was achieved through the PI3K/AKT pathway [13]. Therefore, we aimed to explore the effect of leptin on the IL-17 production by ILC2 in AR in this study.

## 2. Methods

### 2.1. Patients

Fifteen AR patients monosensitized to house dust mite and fifteen healthy controls were enrolled between January 2019 and June 2019 in Guangzhou Women and Children's Medical Center. AR was defined according to Allergic Rhinitis and Its Impact on Asthma Guideline (2010). The criteria included typical symptoms and duration (>2 years), positive allergen test to *Dermatophagoïdes pteronyssinus* and *Dermatophagoïdes farinae* by skin prick test (wheal diameter > 3 mm), and specific IgE measurement (>0.35 kIU/l, Phadia, Uppsala, Sweden) [1]. Patients with atopic dermatitis, asthma, and other nasal diseases and who used systemic and nasal corticosteroids in the previous 1 month were excluded. All the study subjects had normal BMI. The study protocols were approved by local ethics committee boards, and written informed consent was obtained (No. 20691).

### 2.2. Flow Cytometry for ILC2

Peripheral blood mononuclear cells (PBMCs) were isolated from AR using Lymphoprep (Fresenius Kabi Norge AS, Oslo, Norway) density-gradient centrifugation. PMA (50 ng/ml, Sigma-Aldrich), Ionomycin (500 ng/ml, Sigma-Aldrich), and Brefeldin A (BD, Oxford, United Kingdom) were used for restimulation for 4 hours. IL-25 (50 ng/ml), IL-33 (50 ng/ml), TSLP (50 ng/ml), and IL-2 (10 U/ml) (R&D Systems, USA) were also added for ILC2 stimulation. Then, PBMCs were stained with lineage markers (CD2 (RPA-2,10), CD3 (OKT3), CD14 (61D3), CD16 (CB16), CD19 (HIB19), CD56 (TULY56), and CD235a (HIR2), eBioscience, San Diego, CA), FceRI (9E1, eBioscience) and CD45 antibodies (2D1), CRTH2 (BM16, BD Biosciences, NJ), and CD127 antibody (HIL-7R-M21, BD Biosciences, NJ) for identification of ILC2s. IL-5 (JES1-39D10)-, IL-13 (JES10-5A2)-, and IL-17A (BL168)-positive ILC2 cells in PBMCs were determined by intracellular cytokine staining using Cytofix (BD Biosciences, NJ) as described by the manufacturer's instructions. The Beckman flow cytometer machine was used in the test (Beckman Coulter, Hercules, CA, USA).

### 2.3. ILC2 Sorting

After depletion of lineage-positive (Lin^+^) cells by fluorescein isothiocyanate- (FITC-) conjugated antibodies to CD2, CD3, CD14, CD16, CD19, CD56, CD235a, and Fc*ε*RI, lineage-negative (Lin^−^) cells were obtained from PBMCs of AR patients. Then, enriched Lin^−^ cells were stained using phycoerythrin- (PE-) conjugated CRTH2 and hycoerythrin-Cy7-conjugated CD127 (BD Biosciences, NJ). Lin^−^CRTH2^+^CD127^+^ cells (1.5 × 10^5^ cells/ml) were sorted by a FACSAria (BD Biosciences, NJ) with a purity of more than 95% according to instructions. The sorted cells (1.0 × 10^5^) were cultured for 7 days in RPMI-1640 with 10% FBS and 1% penicillin/streptomycin and stimulated by cytokine combination (CC): IL-25 (50 ng/ml), IL-33 (50 ng/ml), TSLP (50 ng/ml), and IL-2 (10 U/ml) (R&D Systems, USA). For stimulation experiments, CC, CC+leptin (100 ng/ml), CC+leptin (100 ng/ml)+ROR*γ*t inhibitor (100 ng/ml), and CC+leptin (100 ng/ml)+Ahr inhibitor (100 ng/ml) (R&D Systems, USA) were added for 72 hours' stimulation in different groups.

### 2.4. Quantitative Real-Time PCR (qRT-PCR)

The total RNA was isolated from ILC2s with TRIzol reagent (Life Technologies, Carlsbad, California). cDNA was synthesized using a cDNA kit (Qiagen). PCR amplification was performed using an ABI PRISM 7300 Detection System. The results were normalized to GAPDH. The primers used in the study were as follows: ROR*γ*t forward 5′-CCG CTG AGA GGG CTT CAC-3′ and reverse 5′-TGC AGG AGT AGG CCA CAT TAC A-3′; Ahr forward 5′-TCCTTGGCTCTGAACTCAAGCTGT-3′ and reverse 5′-GCTGTGGACAATTGAAAGGCACGA-3′; and GAPDH forward 5′-AGCCACATCGCTCAGACAC-3′ and reverse 5′-GCCCAATACGACCAAATCC-3′.

### 2.5. Enzyme-Linked Immunosorbent Assay (ELISA)

The levels of IL-17 in the supernatant of ILC2 were measured using ELISA kits (R&D Systems, USA) as described by the manufacturer. The sensitivity of the assays was as follows: leptin (22 pg/ml) and IL-17 (15 pg/ml).

### 2.6. Statistical Analysis

GraphPad Prism software was used. Statistical significance was determined with the Kruskal-Wallis *H* test or Mann–Whitney *U* test. Spearman rank correlation analysis was done. A *P* value of less than 0.05 was defined as significant difference.

## 3. Results

### 3.1. Correlation between Serum Leptin and IL-17-Producing ILC2s in AR

**Figure 1:**
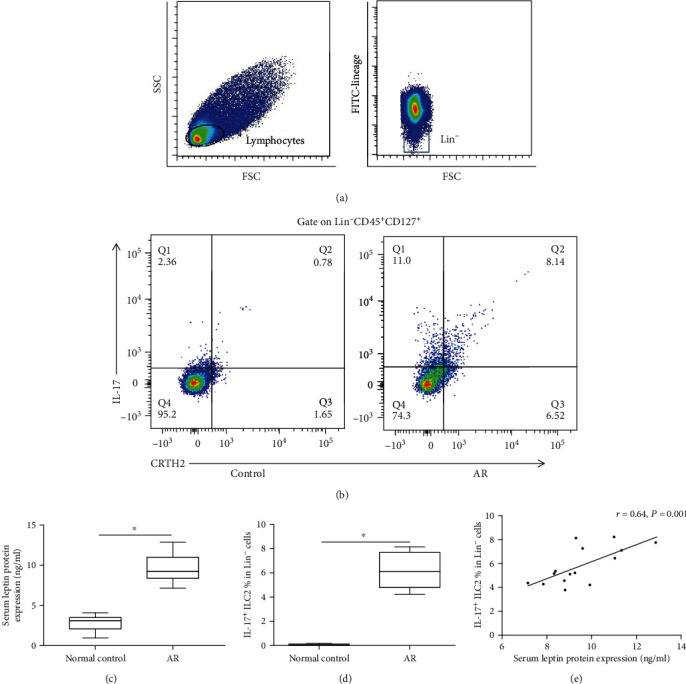
Comparison of serum leptin expression and the frequency of IL-17-producing ILC2s between AR and controls. Gating strategy of ILC2 and representative flow cytometry showed IL-17-producing ILC2 frequency (a, b). The protein expression of serum leptin and the frequency of IL-17-producing ILC2s by ELISA between 15 AR patients and 15 controls (c, d). The correlation between serum leptin and the frequency of IL-17-producing ILC2s in 15 AR patients (e). ^∗^Compared with controls, *P* < 0.05. IL-25 (50 ng/ml), IL-33 (50 ng/ml), TSLP (50 ng/ml), and IL-2 (10 U/ml) were added for 72 hours' stimulation. *P* values were determined by using the Mann–Whitney *U* test. Correlation was determined by using the Spearman rank method.

The characteristics of study subjects are shown in Table 1. The patients between the AR and control groups had comparable age, sex ratio, and age. We found that the frequency of IL-17-producing ILC2s in AR was significantly higher compared with that in controls (*P* < 0.05) (Figures 1(b) and 1(d)). The protein expression of leptin of AR patients was significantly upregulated compared with that of controls (*P* < 0.05) (Figure 1(c)). The serum leptin protein expression was positively correlated with the frequency of IL-17-producing ILC2s in AR patients (*r* = 0.64, *P* < 0.01) (Figure 1(e)).

### 3.2. The Induction of IL-17-Producing ILC2s by Leptin Is Dependent on ROR*γ*t and Ahr

**Figure 2:**
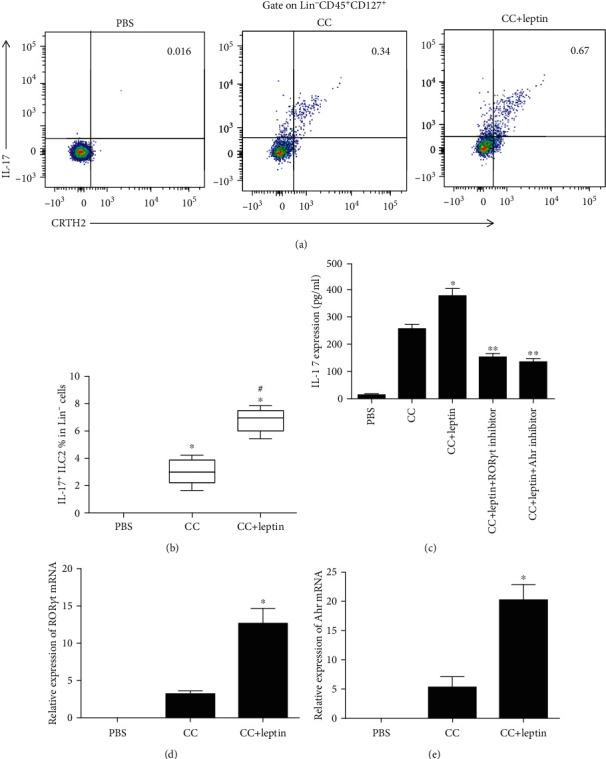
The induction of IL-17-producing ILC2s by leptin is dependent on ROR*γ*t and Ahr in AR patients. (a) The induction of IL-17-producing ILC2s by leptin by flow cytometry. (b) The IL-17 protein production from ILC2 after leptin stimulation by ELISA. (c, d) RT-PCR showed that leptin induced ROR*γ*t and Ahr expression by ILC2. ^∗^Compared with PBS, *P* < 0.05. ^#^Compared with the CC group, *P* < 0.05. CC: cytokine combination: IL-25 (50 ng/ml), IL-33 (50 ng/ml), TSLP (50 ng/ml), IL-2 (10 U/ml). Leptin, 100 ng/ml; ROR*γ*t inhibitor, 100 ng/ml; Ahr inhibitor, 100 ng/ml. PMA (50 ng/ml, Sigma-Aldrich), Ionomycin (500 ng/ml, Sigma-Aldrich), and Brefeldin A (BD, Oxford, United Kingdom) were used for restimulation for 4 hours. Six AR patients were used in this experiment, and three independent tests were performed for every experiment. *P* values were determined by using the Kruskal-Wallis *H* test.

The frequency of IL-17-producing ILC2s from AR patients was almost undetectable when only PBS was added in sorted cells. The addition of CC (IL-25 (50 ng/ml), IL-33 (50 ng/ml), TSLP (50 ng/ml), and IL-2 (10 U/ml)) increased the frequency of IL-17-producing ILC2s. Moreover, the combination of leptin and CC further increased the frequency of IL-17-producing ILC2s compared with CC (Figures 2(a) and 2(b)).

**Figure 3:**
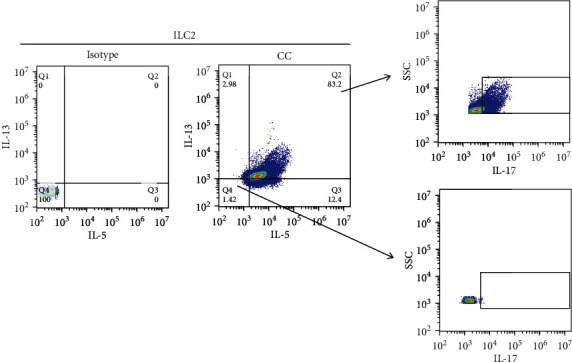
IL-17-producing ILC2s concomitantly express IL-5 and IL-13. The expression of IL-5, IL-13, and IL-17 gated on ILC2 was analyzed by flow cytometry. CC: cytokine combination: IL-25 (50 ng/ml), IL-33 (50 ng/ml), TSLP (50 ng/ml), and IL-2 (10 U/ml). Six AR patients were used in this experiment, and three independent tests were performed for every experiment.

The IL-17 production in the culture supernatant of ILC2 was also upregulated compared with that of controls after leptin stimulation (Figure 2(c)). However, the addition of both ROR*γ*t inhibitor and Ahr inhibitor reduced IL-17 production significantly induced by leptin (Figure 2(c)). Consistently, we observed that leptin induced ROR*γ*t and Ahr expression by ILC2 (Figures 2(d) and 2(e)). To confirm whether IL-17-producing ILC2 had the same features of traditional ILC2 (production of IL-5 and IL-13), we measured the IL-5 and IL-13 expression by IL-17-producing ILC2. Our results suggested that leptin-induced IL-17-producing ILC2 concomitantly expressed IL-5 and IL-13 (Figure 3).

## 4. Discussion

In the present study, we found that leptin-induced IL-17 production from ILC2 cells is dependent on ROR*γ*t and Ahr expression. Our study provided a new pathway in IL-17 regulation.

Leptin, produced by the adipose tissue, plays important roles in immunity [10, 14]. In adaptive immunity, leptin has been proved to exert its action in Th1, Th2, and Th17 response in both humans and mice [15–17]. For example, Zheng et al.'s study found that leptin did not change the differentiation of Th2 cells both in vitro and in vivo but promoted their survival and proliferation, presented as increased expression of IL-4, IL-5, and IL-13 [18]. For innate immunity, our previous data suggest that leptin is also involved in ILC2 regulation in AR and this process was achieved through the PI3K/AKT pathway [13]. Consistently, Zheng et al.'s study showed reduced total number but comparable frequencies of ILC2s between Ob-/- and wild-type control mice of the asthma model.

ILC2 expansion and development need cytokines such as IL-2, TSLP, IL-25, and IL-33. IL-25 promotes inflammatory ILC2 proliferation (Lin^−^IL-17RB^high^KLRG1^high^ST2^−^) in the lung, while IL-33 induced natural ILC2s (Lin^−^IL-17RB^low^KLRG1^int^ST2^+^) [19–21]. A previous study has confirmed that natural ILC2s produce few IL-17, but inflammatory ILC2s can express high levels of IL-17 [19–21]. Inflammatory ILC2s also express ROR*γ*t, which is a master regulator for IL-17 expression in various types of immune cells [22–24].

Our data suggested that the addition of leptin enhanced IL-17+ ILC2 cell expansion and IL-17 production. Moreover, this process is dependent on the expression of ROR*γ*t and Ahr, since the IL-17 expression was significantly inhibited when ROR*γ*t and Ahr inhibitors were added. Ahr is another regulator of the expression of Th17 cytokines, and its receptor (Ahr) is highly expressed by Th17 cells [25].

Previous studies also suggested that Ahr plays important roles in the regulation of ILCs. For example, Ahr is needed for liver-resident ILC1 maintenance and ILC3 maintenance and function [26, 27]. Inhibition of Ahr expression promotes antihelminth immunity in the gut, whereas activation of Ahr suppresses ILC2 function but enhances ILC3 function to protect the host from *Citrobacter rodentium* infection [28]. These results suggested that the Ahr pathway was involved in ILC2-ILC3 balance to mount appropriate immunity against various pathogens. Consistently, our data suggested that leptin regulated IL-17 production by ILC2 through the Ahr pathway.

Consistent with a previous study, our results also suggested that leptin-induced ILC2s concomitantly produce IL-5 and IL-13, which is similar to the memory/effector IL-17+Th2 cells. These data suggest that leptin-induced ILC2 had features of both ILC2 and Th17 cells.

Our study also had some limitations. Firstly, we stimulated blood ILC2 by leptin. But the effects of leptin on local ILC2 (such as the lung and nasal tissue) were not explored. Secondly, the animal models were not used in this study, which also limits our conclusion. Thirdly, the interaction between leptin-induced ILC2 and Th17 cells was not explored.

In summary, our data provide preliminary evidence that leptin-induced IL-17 production from ILC2 cells is dependent on ROR*γ*t and Ahr expression and the blockade of leptin may be a promising target for the treatment of AR.

## Figures and Tables

**Table 1:** Demographic characteristic of AR and control patients.

Groups	AR group	Control
Number	15	15
Sex (male : female)	8 : 7	8 : 7
Age (years)	24.6 (18-42)	27.3 (18-45)
BMI	20.5 (18.7-22.7)	21.2 (19.3-23.4)
TIgE (IU/ml)	178.2 (48.5-671.3)^∗^	38.7 (22.5-58.9)

^∗^Compared with the control group, *P* < 0.05.

## Data Availability

The data used to support the findings of this study are available from the corresponding author upon request.

## References

[1] Brożek J. L., Bousquet J., Baena-Cagnani C. E. (2010). Allergic rhinitis and its impact on asthma (ARIA) guidelines: 2010 revision. *The Journal of Allergy and Clinical Immunology*.

[2] Wang Y., Lobstein T. (2006). Worldwide trends in childhood overweight and obesity. *International Journal of Pediatric Obesity*.

[3] Molet S., Hamid Q., Davoineb F. (2001). IL-17 is increased in asthmatic airways and induces human bronchial fibroblasts to produce cytokines. *The Journal of Allergy and Clinical Immunology*.

[4] Chakir J., Shannon J., Molet S. (2003). Airway remodeling-associated mediators in moderate to severe asthma: effect of steroids on TGF-*β*, IL-11, IL-17, and type I and type III collagen expression. *The Journal of Allergy and Clinical Immunology*.

[5] Ciprandi G., De Amici M., Murdaca G. (2009). Serum interleukin-17 levels are related to clinical severity in allergic rhinitis. *Allergy*.

[6] Quan S. H., Zhang Y. L., Han D. H., Iwakura Y., Rhee C. S. (2012). Contribution of interleukin-17A to the development and regulation of allergic inflammation in a murine allergic rhinitis model. *Annals of Allergy, Asthma & Immunology*.

[7] Wang M., Zhang W., Shang J., Yang J., Zhang L., Bachert C. (2013). Immunomodulatory effects of IL-23 and IL-17 in a mouse model of allergic rhinitis. *Clinical and Experimental Allergy*.

[8] Cai T., Qiu J., Ji Y., Li W., Ding Z., Suo C. (2019). IL-17–producing ST2^+^ group 2 innate lymphoid cells play a pathogenic role in lung inflammation. *Journal of Allergy and Clinical Immunology*.

[9] Golebski K., Ros X. R., Nagasawa M. (2019). IL-1*β*, IL-23, and TGF-*β* drive plasticity of human ILC2s towards IL-17-producing ILCs in nasal inflammation. *Nature Communications*.

[10] Matarese G., Moschos S., Mantzoros C. S. (2005). Leptin in immunology. *Journal of Immunology*.

[11] Yu Y., Liu Y., Shi F. D., Zou H., Matarese G., La Cava A. (2013). Cutting edge: leptin-induced ROR*γ*t expression in CD4^+^T cells promotes Th17 responses in systemic lupus erythematosus. *Journal of Immunology*.

[12] Deng J., Liu Y., Yang M. (2012). Leptin exacerbates collagen-induced arthritis via enhancement of Th17 cell response. *Arthritis and Rheumatism*.

[13] Zeng Q., Luo X., Tang Y., Liu W., Luo R. (2020). Leptin regulated ILC2 cell through the PI3K/AKT pathway in allergic rhinitis. *Mediators of Inflammation*.

[14] Fantuzzi G., Faggioni R. (2000). Leptin in the regulation of immunity, inflammation, and hematopoiesis. *Journal of Leukocyte Biology*.

[15] Batra A., Okur B., Glauben R. (2010). Leptin: a critical regulator of CD4^+^ T-cell polarization *in vitro* and *in vivo*. *Endocrinology*.

[16] Youssef D. M., Elbehidy R. M., Shokry D. M., Elbehidy E. M. (2013). The influence of leptin on Th1/Th2 balance in obese children with asthma. *Jornal Brasileiro de Pneumologia*.

[17] Liu W., Zeng Q., Zhou L., Li Y., Chen Y., Luo R. (2018). Leptin/osteopontin axis contributes to enhanced T helper 17 type responses in allergic rhinitis. *Pediatric Allergy and Immunology*.

[18] Zheng H., Zhang X., Castillo E. F., Luo Y., Liu M., Yang X. O. (2016). Leptin enhances TH2 and ILC2 responses in allergic airway disease. *Journal of Biological Chemistry*.

[19] Huang Y., Guo L., Qiu J. (2015). IL-25-responsive, lineage-negative KLRG1^hi^ cells are multipotential ‘inflammatory’ type 2 innate lymphoid cells. *Nature Immunology*.

[20] Huang Y., Paul W. E. (2016). Inflammatory group 2 innate lymphoid cells. *International Immunology*.

[21] Zhang K., Xu X., Pasha M. A. (2017). Cutting edge: notch signaling promotes the plasticity of group-2 innate lymphoid cells. *Journal of Immunology*.

[22] Ivanov I. I., McKenzie B. S., Zhou L. (2006). The orphan nuclear receptor ROR*γ*t directs the differentiation program of proinflammatory IL-17^+^ T helper cells. *Cell*.

[23] Kanai T., Mikami Y., Sujino T., Hisamatsu T., Hibi T. (2012). ROR*γ*t-dependent IL-17A-producing cells in the pathogenesis of intestinal inflammation. *Mucosal Immunology*.

[24] Eberl G. (2017). ROR*γ*t, a multitask nuclear receptor at mucosal surfaces. *Mucosal Immunology*.

[25] Kimura A., Naka T., Nohara K., Fujii-Kuriyama Y., Kishimoto T. (2008). Aryl hydrocarbon receptor regulates Stat 1 activation and participates in the development of Th17 cells. *Proceedings of the National Academy of Sciences of the United States of America*.

[26] Zhang L. H., Shin J. H., Haggadone M. D., Sunwoo J. B. (2016). The aryl hydrocarbon receptor is required for the maintenance of liver-resident natural killer cells. *The Journal of Experimental Medicine*.

[27] Kiss E. A., Vonarbourg C., Kopfmann S. Natural aryl hydrocarbon receptor ligands control organogenesis of intestinal lymphoid follicles. *Science*.

[28] Li S., Bostick J. W., Ye J. (2018). Aryl hydrocarbon receptor signaling cell intrinsically inhibits intestinal group 2 innate lymphoid cell function. *Immunity*.

